# Effect of Deep Cryogenic Treatment on Wear and Galling Properties of High-Speed Steels

**DOI:** 10.3390/ma14247561

**Published:** 2021-12-09

**Authors:** Patricia Jovičević-Klug, Marko Sedlaček, Matic Jovičević-Klug, Bojan Podgornik

**Affiliations:** 1 Institute of Metals and Technology, Lepi pot 11, 1000 Ljubljana, Slovenia; bojan.podgornik@imt.si; 2 Jožef Stefan International Postgraduate School, Jamova cesta 39, 1000 Ljubljana, Slovenia; marko.sedlacek@imt.si; 3 Max-Planck-Institute für Eisenforschung, Max-Planck-Straße 1, 40237 Düsseldorf, Germany; m.jovicevic-klug@mpie.de

**Keywords:** deep cryogenic treatment, high speed steel, wear, galling, impact loading

## Abstract

New approaches to improving wear resistance with an affordable and noncomplex technology, such as deep cryogenic treatment, (DCT0), are receiving attention. The aim of this study is to investigate the effect of DCT on the friction and wear performance of high-speed steels. AISI M2, AISI M3:2 and AISI M35 were heat-treated under different conditions, and then investigated under dry sliding conditions. Tribological testing involved different contact conditions, prevailing wear mechanisms and loading conditions. The DCT effect on sliding wear resistance depends on HSS steel grade, as well as contact conditions and wear mode, whereas it improves the dynamic impact of the wear and galling resistance.

## 1. Introduction

In tool industry high-speed steels (HSS), such as AISI M2; AISI M4 and AISI M42, are most commonly used for cutting tools (saw blades, knives, drill bits, etc.). HSS are preferred materials for cutting tools, as they have a high hardness (above 60 HRC), high wear resistance and high thermal resistance [1]. The tool’s performance is determined not only by the material’s intrinsic properties, but also by the surface properties, including friction, abrasive and adhesive wear resistance, galling resistance and resistance to mechanical and thermal cracking, i.e., fatigue. In addition, tools can be exposed to very demanding and complex loading conditions (chemical, mechanical, thermal, tribological, etc.). As a consequence, different approaches to improving tool properties and increasing tool performance for a prolonged lifecycle are applied, such as heat treatment, thermo-chemical treatments, surface texturing and coatings [1].

The easiest way to manipulate steel properties and performance is to change the heat treatment parameters (austenitizing and tempering temperature, quenching speed, etc.) to induce microstructural changes and, in turn, the mechanical, tribological and corrosion properties of the processed steel [2]. Another method for modifying the microstructure is the introduction of additional processing of the material, such as deep cryogenic treatment (DCT). DCT is used as a supplementary processing method and was proven to be effective in prolonging tool life by changing the material’s mechanical properties (hardness, fracture toughness, compressive and tensile strength, impact toughness, etc.) [3]. During DCT, the material is exposed for certain time to temperatures below −160 °C, which influences the microstructure development. In steels, it induces more homogenous carbides distribution, a higher precipitation of carbides and matrix change (increased amount of martensite and reduction in retained austenite) [4]. However, DCT performance is dependent on the selected soaking temperature, soaking time, cooling and heating rate and placement of DCT within the heat treatment process (before or after tempering) [5].

The literature review on DCT-treated HSS in relation to tribological properties, including wear resistance, showed that the majority of studies concentrated on AISI M2, as the most commonly used HSS. Leskovšek et al., 2006 [6], Pellizzari et al., 2008 and 2012 [7,8,9], Fantineli et al., 2020 [10] and Zhou et al., 2020 [11] clearly stated that DCT has a positive effect on the abrasive wear resistance of AISI M2 steel, whereas studies on other HSS are rather limited and show contradictory results in terms of wear resistance change after DCT. This is proposed to be a consequence of different microstructural changes (carbide precipitation, carbide distribution and characteristics of the matrix), which then influences the material’s mechanical and tribological properties [6,7,8,9]. Nevertheless, most cases indicate DCT to have a positive effect [4,12,13]; some negative effects on wear resistance were also reported [8]. Most of the research is focused on abrasive wear resistance, while resistance to adhesive wear, galling and dynamic impact wear has rarely been investigated, not only in connection to DCT, but also more generally in connection to selected HSS. Furthermore, it is known that steel type, composition and heat treatment conditions all determine the impact and effectiveness of DCT on changes in microstructure, which consequently influences the changes in the materials’ properties [7,14,15,16]. To overcome the contradictory results and to acquire a clear understanding of DCT impact on HSS wear and tribological properties, testing results must be correlated to microstructural changes induced by DCT.

For the above reasons, the aim of this study is (1) to explore the effect of deep cryogenic treatment (DCT) on the wear and galling resistance of three different high-speed steels of the same type (Mo-type), but with a different ratio of alloying elements (C, V, Co) that are manufactured by different manufacturing technologies (wrought/powder-metallurgy (P/M)). The next aim is (2) to test the influence of the chemical composition of HSS in relation to different heat treatments (higher/lower austenitization temperature and lower/higher tempering temperature) and how this reflects on the tribological properties. (3) In order to provide deeper understanding of the DCT mechanism on wear and galling resistance, testing under different contact and loading conditions to provoke different wear mechanisms was performed and evaluated.

## 2. Materials and Methods

### 2.1. Materials and Heat Treatment

All three high-speed steels, AISI M2 (M.N. 1.3343, EN HS6-5-2, wrought steel), AISI M3:2 (M.N. 1.3395, EN HS6-5-3, P/M steel) and AISI M35 (M.N. 1.3243, EN HS6-5-2-5, wrought steel), were obtained in the form of rolled, peeled and soft annealed rods, from the following producers: Sij Ravne, Ravne, Slovenia (AISI M2 and AISI M35) and HSM, Georgensgmuend, Germany (AISI M3:2). Samples for specific testing were then machined from these rods: abrasive/adhesive wear and dynamic impact wear test discs (Ø 20.0 × 8.0 mm), and test cylinders for galling tests (Ø 10.5 × 100.0 mm). After heat treatment all samples were also surface polished down to Ra = 0.05 µm (the surface roughness was the same for both heat treatments regimes and for all three steel grades), and then subjected to heat treatment. The measured chemical composition and selected mechanical properties of each steel grade is provided in Table 1.

For all three steel grades, two sets of heat treatment parameters were used to evaluate the effect of selected austenitizing/tempering temperatures. First set of specimens (A1–A2, B1–B2, C1–C2) was hardened at upper austenitizing temperature, as recommended by the steel producer, and then tempered at lower temperature in order to promote higher hardness over toughness. The second set (A3–A4, B3–B4, C3–C4) was hardened at lower austenitizing temperature and tempered at higher tempering temperature, therefore promoting a higher toughness over hardness. Each set from the individual steel group was austenitized and quenched in a single step in a horizontal vacuum furnace Ipsen VTTC324-R. Quenching was performed with N_2_ gas at a pressure of 5 bars with an average quenching rate of 7–8 °C/s. After hardening, one group of specimens from each steel and heat treatment set was conventionally heat-treated (CHT) (A1, A3, B1, B3, C1, C3) (control group), comprising of triple tempering according to a steel producer recommendation. The second group was subjected to DCT, which was performed immediately after quenching by gradual immersion (approximate cooling rate of 10 °C/s) of the samples (A2, A4, B2, B4, C2, C4) in liquid nitrogen for 24 h, followed by a single tempering cycle. Heat treatment parameters of each set are given in Table 2.

### 2.2. Testing Methods

#### 2.2.1. Reciprocating Sliding Tests

**Figure 1:**
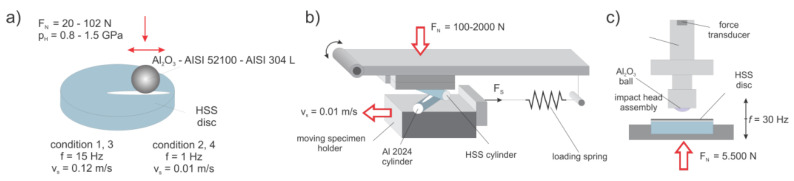
Schematic representation of different testing methods. (**a**) Reciprocating sliding: ball-on-flat contact configuration. (**b**) Cross-cylinder galling test. (**c**) Dynamic impact wear testing.

Abrasive/adhesive wear resistance and coefficient of friction were determined under reciprocating dry sliding conditions using ball-on-flat contact configuration (Figure 1a) with a stroke length of 4 mm. The testing was performed with 3 different wear mechanism conditions typically found in cutting by utilizing different counter bodies (all of the same size; Ø 20 mm): Al_2_O_3_ (~1750 HV), favoring abrasive wear; AISI 52100 (100Cr6; ~700 HV) favoring a combination of abrasive/adhesive wear; and AISI 304L (~300 HV), favoring pure adhesive wear. Four different loading conditions were applied, using two loads and two sliding speeds (high sliding speed/high load, low sliding speed/high load, high sliding speed/low load and low sliding speed/low load). High-load conditions corresponded to contact pressure of 1.5 GPa (Al_2_O_3_ ball) and 1.0 GPa (AISI 52100 and 304L ball), obtained by applying load of 102 N and 40 N, respectively. Whereas for low load conditions, contact pressures of 1.0 GPa (Al_2_O_3_) and 0.8 GPa (AISI 52100 and 304L) were achieved with the application of a load of 40 N and 20 N, respectively. The high sliding speed of 0.12 m/s was achieved at a frequency of 15 Hz and the low sliding speed of 0.01 m/s at frequency of 1 Hz. The summary of all conditions is presented in Table 3. Dry sliding wear and friction tests were performed at room conditions, with a total sliding time and distance of 7500 s/60 m (low sliding speed) and 1667 s/200 m (high sliding speed). The measurements were performed at least 3 times for each condition and sample group.

The wear volume of the HSS disc samples was measured using 3D confocal focus variation microscope (Alicona InfiniteFocus, Raaba/Graz, Austria) and the specific wear rate [19] was calculated afterwards based on Equation (1), where *W* is wear volume in mm^3^, *F_N_* is normal load in N, and *S* is sliding distance in m:

(1) $$k=\frac{W}{F_{N}\times S}\;\left[\frac{{\mathrm{mm}}^{3}}{\mathrm{Nm}}\right]$$

The wear scars were also investigated with scanning electron microscope (SEM; JSM-6500F, Jeol, Tokyo, Japan), to identify/confirm prevailing wear mechanisms and wear related failures and defects. Coefficient of friction (COF) was recorded continuously during sliding, and then the average value for the steady–state conditions (800–1666 s for sliding speed of 0.12 m/s and 500–7500 s for sliding speed of 0.01 m/s) was determined for all three counter-body conditions.

#### 2.2.2. Galling

The effect of material resistance against galling, defined as an accumulation of work material on the tool surface and commonly observed in forming operations such as deep drawing and extrusion, was evaluated under progressively increasing loading conditions by a cross-cylinder load-scanning test rig (Figure 1b) [17], thus simulating metal forming. Al2024 cylinders in a T6 condition (Ø10 mm × 100 mm; 155 HB) were used as a moving counter-body, representing one of many very difficult materials in cold form. All Al specimens were polished and cleaned with ethanol to achieve clean and equal surface condition (Ra = 0.1 µm) for all tests. Galling tests, repeated three times for each tool steel specimens group, were performed dry, using sliding speed of 0.01 m/s and normal load from 100 N to 2000 N, resulting in severe plastic deformation of the Al cylinder during testing. For determining the critical loads for galling initiation and gross galling (transfer layer) formation, the wear tracks after sliding were analyzed using 3D confocal focus variation microscope (Alicona InfiniteFocus, Raaba/Graz, Austria) at specific loading regions, which were selected based on the coefficient of friction evolution during sliding [20]. All friction data were processed with the LOESS (locally weighted smoothing) method with a 0.06-point span.

#### 2.2.3. Dynamic Impact Wear

In order to investigate the dynamic impact wear properties, servo-hydraulic dynamic testing machine Instron 8802, Norwood, MA-USA was used. Tests were performed in such a way that the HSS disc samples were constantly impacted against a static alumina ball (Al_2_O_3_) at an impact frequency of 30 Hz (Figure 1c). Test was regulated through the monitoring of the impact force, which varied in a sinusoidal form with a compressive force peak of 5.5 kN, and corresponded to a contact pressure of 3.5 GPa. At maximum displacement amplitude, the ball and the test sample were completely separated with a 0.5 mm gap. Tests were limited to 300,000 cycles and performed at normal room conditions (T = 23 ± 3 °C; RH = 50 ± 10%). In order to prevent adhesion of wear debris to the ball, the steel disc contact surface was lubricated with lithium grease before the experiment. For each specimen, the test was repeated at least three times and for each test a fresh Ø 32 mm Al_2_O_3_ ball was used. After testing, the wear volume of the steel sample was measured using 3D confocal focus variation microscope (Alicona InfiniteFocus, Raaba/Graz, Austria).

#### 2.2.4. Microstructure of Selected High-Speed Steels

Microstructure analysis and explanation of the possible mechanism related to microstructure of all three HSS (AISI M2, AISI M3:2 and AISI M35) is based on our previous studies by Jovičević-Klug et al., 2020 [16] and Jovičević-Klug et al., 2021 [21]. The main finding of microstructure analysis of all three HSS showed that the matrix is lath martensite, whereas retained austenite (RA) was found in all three cases < 1 vol. %. The carbides present in all three steels, independent of the heat treatment regimens, are MC (M = V), M_6_C (Mo, W, Fe), M_2_C (Mo, V, W) and M_23_C_6_ (Cr, Fe). DCT increases the precipitation of carbides, induces the size reduction in carbides, and reduces amount of RA; martensitic laths are smaller and have preferable orientated along [101] and [001] directions, and oxidation state of DCT samples is changed.

## 3. Results

### 3.1. Sliding Wear Resistance

#### 3.1.1. Conditions Favoring Abrasive Wear (Al_2_O_3_ Counter-Body)

**Figure 2:**
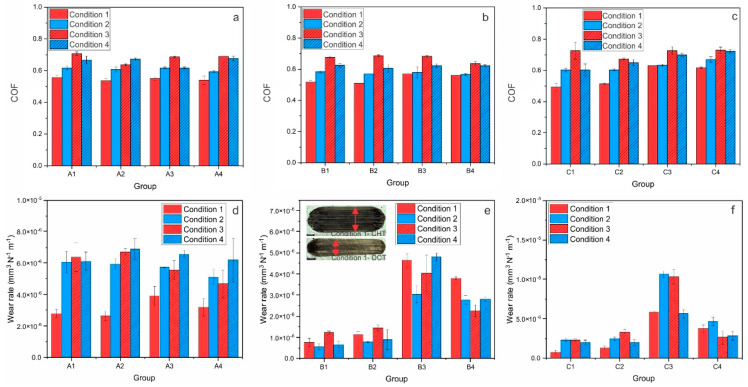
Results of abrasive wear conditions (Al_2_O_3_ counter-body) for all three steels; A (AISI M2), B (AISI M3:2) and C (AISI M35), where (**a**–**c**) is steady-state coefficient of friction for each condition and (**d**–**f**) is wear rate for each steel at certain condition. (**e**) the insert shows the average representation of wear scar for all three steels of conventionally (CHT) and deep cryogenically heat-treated samples; the black scale represents 500 µm.

COF and wear rate (k) results for conventionally and deep cryogenic heat-treated HSS (AISI M2, AISI M3:2 and AISI M35) tested under reciprocating dry sliding conditions against Al_2_O_3_ ball, which favors abrasive wear, are shown in Figure 2. Under the abrasive wear conditions used in this investigation, the steady-state COF is generally independent of the heat treatment process (CHT or DCT) and HSS grade. However, it changes depending on the sliding condition (load and sliding speed), as shown in Figure 2a–c. Under high-load/high sliding speed, COF is in the range of 0.5–0.55, except for steel C that is hardened from the lower austenitizing temperature (C3, C4), where it increases to 0.6. By switching to high load/low sliding speed conditions, COF increases to ~0.6 and, for low load/high sliding speed, increases to ~0.7. For low load/low sliding speed, COF decreases slightly to ~0.65, while for steel C, hardened from the lower austenitizing temperature (C3, C4), it again shows slightly higher values of 0.7. In terms of abrasive wear resistance (Figure 2d–f), the first observation is that for all three HSS wear scars of DCT samples (Figure 2d) are narrower compared to CHT samples (Figure 2d) by roughly 25%. For steel A, wear rates are in the range of 2.0 × 10^−6^–7.0 × 10^−6^ mm^3^/Nm. If hardened from high austenitization temperature and followed by a low tempering temperature (promoted hardness; A1, A2), DCT does not show any noticeable effect, except at low load/low sliding speed conditions, under which approximately a 10% increase in wear rates can be observed (Figure 2d). On the other hand, when hardened from low austenitization temperature (promoting toughness; A3, A4), generally DCT improves abrasive wear resistance of steel A regardless of the sliding condition (up to 25%). In the case of steel B (k = 0.5 × 10^−6^–5.0 × 10^−6^ mm^3^/Nm), DCT generally improves its abrasive wear resistance under low austenitization temperature and high tempering temperature regardless of the sliding conditions, with the improvement being in the range of 30–60%, especially prominent for low loading conditions (Figure 2e). However, under all sliding conditions in combination with high austenitization temperature and low tempering temperature, DCT results in reduced abrasive wear resistance (up to 20%). Finally, for steel C (k = 1.0 × 10^−6^–11.0 × 10^−6^ mm^3^/Nm) there is a general trend of negligible to only minor negative effect on abrasive wear resistance, when applying DCT treatment in combination with high austenitization temperature and low tempering temperature (Figure 2f). Whereas with low austenitization temperature and high tempering temperature, DCT improves the wear resistance regardless of sliding condition, up to 50%.

#### 3.1.2. Conditions Favoring Combination of Abrasive and Adhesive Wear (AISI 52100 Counter-Body)

**Figure 3:**
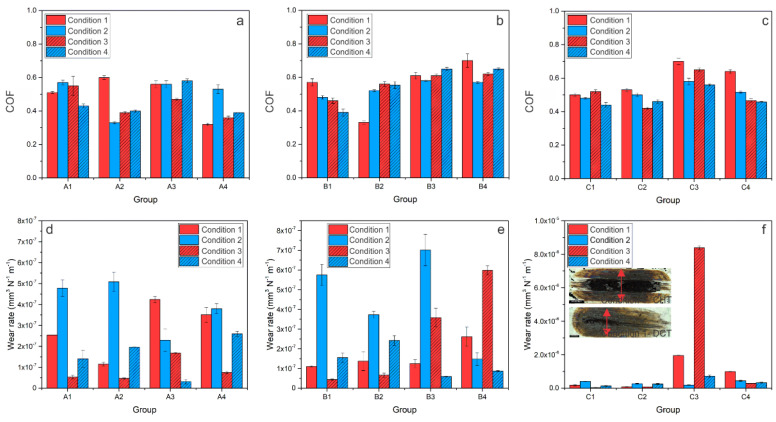
COF and wear rate results for contact conditions favoring a combination of abrasive and adhesive wear for all three steels, A (AISI M2), B (AISI M3:2) and C (AISI M35), where (**a**–**c**) is steady-state coefficient of friction for each condition and (**d**–**f**) is the wear rate for each steel at certain condition. (**f**) the insert presents the average representation of wear scar for all three steels of conventionally (CHT) and deep cryogenically heat-treated specimens; the black scale represents 500 µm.

Analysis of the wear scars under the combination of abrasive/adhesive wear (AISI 52100 counter-body) showed a similar trend, as observed for pure abrasive wear conditions, with wear scars of DCT samples being narrower compared to CHT samples by about 15%. COF and wear rate results for CHT- and DCT-treated HSS tested under the mixed condition of abrasive and adhesive wear are shown in Figure 3. In general, for steel A, COF decreased from 0.5–0.6 (CHT sample) to 0.3–0.4 after DCT, which is a reduction of 20–25% (Figure 3a). Steel B shows no significant changes in COF. However, for high austenitizing temperature groups (B1-B2), CHT group (B1) has a decreasing and the DCT group has an increasing COF trend as the contact conditions intensify, as shown in Figure 3b. The origin of such behavior could be in different prevailing wear mechanisms for DCT and CHT samples. Steel C (Figure 3c) also shows different trends depending on the heat treatment. There is no significant difference in COF between the CHT and DCT group for a higher austenitizing and lower tempering temperature (C1-C2). However, for the second heat treatment regime of a lower austenitizing and higher tempering temperature, the DCT samples have about 20–25% lower COF (0.5–0.6) as compared to CHT samples (0.6–0.7), similar to that observed for steel A. With regard to wear resistance (Figure 3d–f), involving a combination of abrasive and adhesive wear, steel A wear rates are in the range of 0.5–5.0 × 10^−7^ mm^3^/Nm (Figure 3d). In this case, (steel A) DCT has a positive effect on wear resistance for high sliding speed conditions (contact conditions 1 and 3), but detrimental under low-speed conditions (contact conditions 2 and 4), regardless of the austentizing and tempering temperature. Steel B wear rate results are in range of 0.5–7.0 × 10^−7^ mm^3^/Nm, with DCT generally resulting in a negative trend. The only exceptions are high load and low sliding speed conditions (Figure 3e). Finally, for steel C, wear rates are in the range of 0.1–85 × 10^−7^ mm^3^/Nm, for which a generally positive trend of improved wear resistance is observed for DCT samples, especially when combined with lower austenitizing and higher tempering temperature (Figure 3f), similar to the conditions favoring abrasive wear (Figure 2f).

#### 3.1.3. Conditions Favoring Combination of Abrasive and Adhesive Wear (AISI 304L Counter-Body)

**Figure 4:**
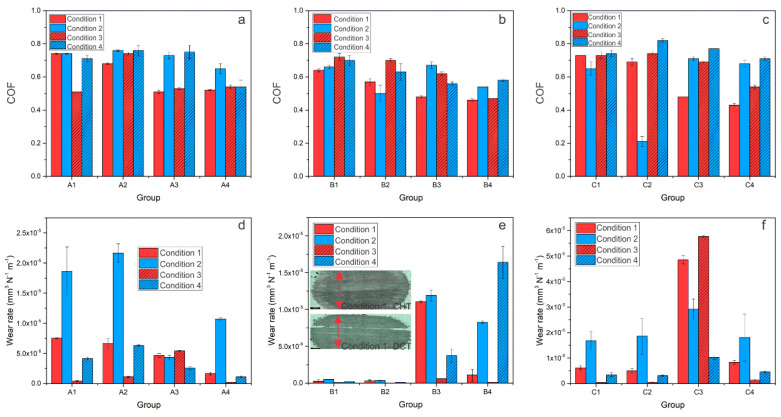
COF and wear rate results for contact conditions favoring adhesive wear for all three steels A (AISI M2), B (AISI M3:2) and C (AISI M35), where (**a**–**c**) is a steady-state coefficient of friction for each condition and (**d**–**f**) is wear rate for each steel at a certain condition. (**e**) The insert shows the average representation of the wear scars for all three steels of conventionally (CHT) and deep cryogenically heat-treated samples, the black scale represents 500 µm.

Additionally, in the case of a softer counter-body (AISI 304L) favoring adhesive wear, wear scars for DCT samples are generally narrower when compared to their CHT counterparts, although not by more than 10%. The COF results of steel A show no significant difference between DCT and CHT treatments (COF is between 0.4–0.6). The only noticeable exception is at the low sliding speed conditions and low austenitizing temperature, with the DCT samples displaying a 20–25% lower steady-state friction (Figure 4a). Steel B and C (Figure 4b,c) show a general trend of COF improvement with DCT when adhesive wear prevails. Adhesive wear resistance for steel A (k = 0.1–2.25 × 10^−5^ mm^3^/Nm) improves with DCT (up to 35%) for high sliding speed conditions (condition 1 and 3), while it deteriorates under slow sliding speed conditions (conditions 2 and 4; Figure 4d). In the case of steel B, DCT generally has a positive trend, improving adhesive wear resistance by 10–65% (k = 0.1–1.6 × 10^−5^ mm^3^/Nm). The only exception is a low austenitizing temperature and low load/low sliding speed condition (condition 4) (Figure 4e). For steel C, the adhesive wear rate is in the range of 0.1–5.5 × 10^−5^ mm^3^/Nm. While, for high austenitizing and low tempering temperatures, DCT has no evident effect, it significantly improves adhesive wear resistance (by up to 90%) when combined with low austenitizing and high tempering temperatures (Figure 4f).

### 3.2. Galling

**Figure 5:**
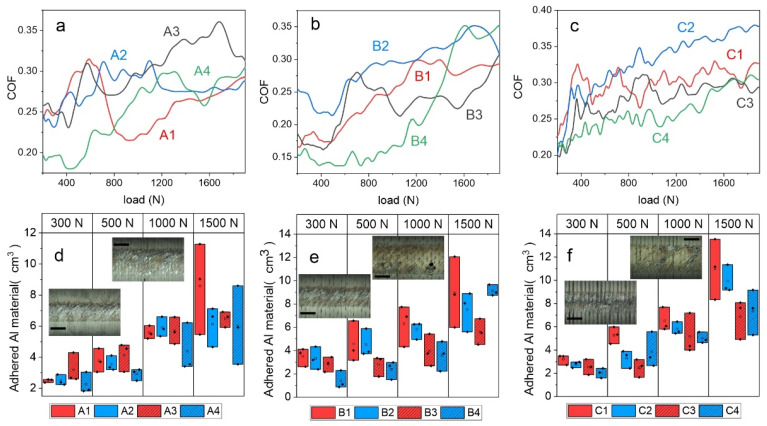
Resulting deposited volume of aluminum material from the counter body at specific normal loads for all heat-treated states of steels (**a**) A, (**b**) B and (**c**) C. Within the diagrams, exemplar images of areas at different loads displaying complete adhesion of counter body material, and a region indicating delamination of adhered material, are provided. In (**d**–**f**), the representative friction coefficient (COF) profiles versus applied load of each heat-treated state of steels, A, B and C, are presented, respectively. (**d**–**f**) The inserts show the average representation of galling for all three steels of conventionally (CHT) and deep cryogenically heat-treated samples; the black scale represents 500 µm.

The galling test results (Figure 5a–f) generally show a positive trend of DCT on galling initiation and Al transfer layer formation on HSS. The results were evaluated by monitoring the coefficient of friction (COF) as a function of the load and identifying abrupt changes in friction, indicating galling initiation and transfer layer formation [17]. (Figure 5a–c). Furthermore, the amount of transferred and adhered Al material to the HSS contact surface at four different loads (300 N, 500N, 1000 N and 1500 N; Figure 5d–f) is determined to evaluate the galling processes. The results of steel A (AISI M2) (Figure 5a) show an abrupt increase in COF already, at low loads of about 400 N and 500 N, when conventionally heat treated from higher (A1) and lower (A3) austenitization temperatures, respectively, followed by a high and unstable COF due to stochastic deposition and the removal of Al material. The application of DCT results in postponed friction increase to higher load values of 700 N (A2) and 1000 N (A4). However, when analyzing the adhered amount of Al material to the steel A cylinder, DCT does not show any improvement if combined with a high austenitizing temperature and low tempering temperature, at least not before the extensive plastic deformation of Al counter cylinders takes place (1500 N). However, for a low austenitization temperature and high tempering temperature (A3–A4) the amount of adhered Al material was reduced by 30% with DCT, which is observed across a broad load range (300–1500 N). For steel B (AISI M3:2) (Figure 5e), the increase in friction takes place between 600 N and 800 N when conventionally heat treated (B1, B3), at which a low austenitization temperature and high tempering temperature (B3) results in a 15–40% smaller amount of transferred Al material. Similar to steel A, the inclusion of DCT provides an improved galling resistance, but only when combined with lower austenitization and higher tempering temperature (B4). In this case, an abrupt increase in friction, indicating gross galling, is postponed to 1200 N and the amount of adhered material in the low-to-mid load range (300–1000 N) is reduced by ~20% as compared to CHT counterpart (B3), as shown in Figure 5b,e.

In the case of steel C, the coefficient of friction shows a similar trend for all four heat treatment regimes (Figure 5f). In all cases, COF steadily increases with load, with galling initiation already indicated at a high value of approximately 300 N. With a low austenitizing temperature and high tempering temperature (C1-C2), the friction values are lower compared to the other heat treatment procedure (C3-C4), whether combined with DCT or not. In terms of adhered Al material, DCT provides up to a 50% improvement in galling resistance when combined with a high austenitizing/low tempering temperature (C2), while its beneficial effects are more or less lost for the other case (low austenitizing/high tempering temperature; C4).

### 3.3. Dynamic Impact Wear

**Figure 6:**
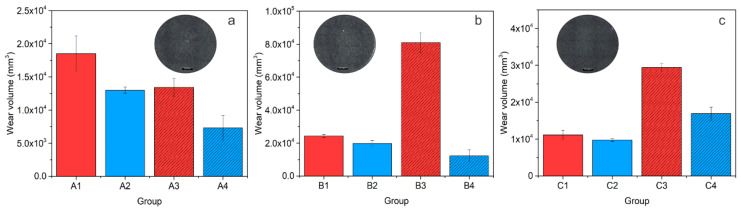
Dynamic impact wear (DIW) as a function of heat treatment for all three steels, where A stands for AISI M2 (**a**), B for AISI M3:2 (**b**) and C for AISI M35 (**c**). (**a**–**c**) Insert photos show the average representation of wear scar for each steel grade for conventionally (CHT) and deep cryogenically heat-treated samples; the black scale represents 500 µm.

In Figure 6, the dynamic impact wear (DIW) properties of the investigated steels, A (AISI M2), B (AISI M3:2) and C (AISI M35), are presented in the form of disc wear volume as a function of heat treatment. In the case of steel A, the dynamic impact wear volume is between 0.6 × 10^4^ mm^3^ and 1.8 × 10^4^ mm^3^, and is lowered by using a lower austenitization temperature (A3) and/or DCT (A2, A4) by 30–40%, as shown in Figure 6a. Dynamic impact wear results for material B are presented in Figure 6b. It can be seen that for steel B, DCT has a positive effect on DIW resistance, especially when DCT is combined with a low austenitization temperature and high tempering temperature (B3, B4). In this case, the dynamic impact wear volume of steel B is reduced from 8.0 × 10^4^ mm^3^ to 1.0 × 10^4^ mm^3^, which is lower than for both CHT and DCT samples heat treated from a high austenitization and low tempering temperature, as shown in Figure 6b. In the case of steel C, DCT has only a minor improvement effect (~10%) on steel DIW resistance when high austenitization and low tempering temperatures are applied, as shown in Figure 6c. By using low austenitization and high tempering temperatures, the DIW properties of steel C are more strongly improved with DCT, reducing the wear volume from ~3 × 10^4^ mm^3^ to ~1.75 × 10^4^ mm^3^.

## 4. Discussion

### 4.1. Sliding Wear Resistance (Abrasive and Adhesive Wear)

Detailed microstructure analysis of the investigated HSS after CHT and DCT is presented in Jovičević-Klug et al., 2020 [16] and Jovičević-Klug et al., 2021 [21]. As described, for all three HSS steels, the matrix is composed of lath martensite (hardness of 53–63 HRC), with the retained austenite (RA) being under 1 vol. %. In addition, carbides in the form of MC (enriched with V; hardness of 2500–3000 HV [22]), M_6_C (enriched with W, Mo and Fe; hardness of 1100–1700 HV [23]) and M_23_C_6_ (enriched with Fe and C; hardness of 1000–1450 HV [24]) are observed (Supplementary Material Table S1). However, the number of carbides is higher in DCT samples compared to CHT samples, which mostly originates from the increased number of precipitated M_23_C_6_ carbides (30% higher volume fraction and up to 140% increased number). Additionally, after DCT, the microstructure is more homogenous and the average size of carbides is up to 20% smaller compared to CHT counterparts [16]. Aside from DCT, the heat treatment regime (austenitizing and tempering temperature) also defines the number of precipitated carbides, with a lower austenitizing and higher tempering temperature resulting in up to a 40% higher number of precipitated carbides. The increased number of MC carbides is observed for steel B (AISI M3:2) and M_6_C for steels A (AISI M2) and C (AISI M35). In this case, the application of DCT increases the volumetric fraction of carbides (mostly M_23_C_6_ carbides) for steels A and B, whereas, for steel C, the volumetric fraction of carbides remains similar, although is finer after DCT. The increased precipitation of finer carbides, such as M_23_C_6_, can improve wear resistance by the overall hardening of the material. Aside from this, DCT-induced microstructural changes also resulted in changes in mechanical properties (proven in the study by Jovičević-Klug et al., 2021 [17]), including increased hardness and toughness.

**Figure 7:**
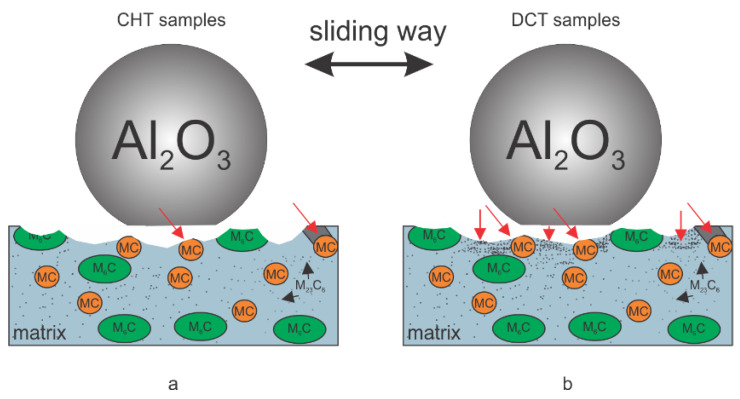
Schematic representation of sliding dynamics during sliding ball-on-flat contact testing for (**a**) conventional heat-treated samples and (**b**) deep cryogenic heat-treated samples. The red arrows indicate the movement of carbides and the agglomeration of them on the wear surface during the sliding test.

In terms of the abrasive wear resistance (Figure 7a,b), in general DCT has no dominant effect for heat treatment conditions favoring high hardness (high austenitizing/low tempering temperature), with the matrix hardness being the dominant factor. However, more homogeneous and finer carbides distribution with reduced agglomeration may even result in minor reduction of abrasive wear resistance after DCT. However, under conditions favoring higher toughness (low austenitizing/high tempering temperature) DCT improves abrasive wear resistance of HSS steels for all contact conditions, especially for steel B and C, also displaying considerable increase in hardness. The general tendency of more pronounced effect of DCT on abrasive wear resistance at lower austenitizing and higher tempering conditions is associated with the softer matrix, higher precipitation of carbides and higher toughness, also typical for PM steels. In this case, DCT promotes increased precipitation of finer carbides and their more homogeneous distribution, facilitating improvement in toughness as well as hardness (Table 2). It is proposed that the increased toughness allows the localized deformation of the softened matrix material due to high contact temperatures, which in turn allows displacement of the carbides on the contact surface and deeper into the matrix (indicated by vertical red arrows in Figure 7b), protecting the matrix from the nominal friction forces and wear. In turn, the initial removal of material mostly results from the accommodation of this effect, which is then considerably reduced with intensified agglomeration of carbides over time. At low sliding speeds absence of matrix softening could promote carbides pull-out (mostly MC carbides, as also observed by Pellizzari et al., 2012 [9]) and microcracking (M_23_C_6_ and M_6_C) which may act as third body particles and causing microploughing, thus resulting in increased wear rate at increased carbides precipitation by DCT. This effect strongly depends on contact conditions and the size and shape of carbides. Smaller and more uniform, smaller is the negative effect, thus switching favor toward DCT, as observed in our case (see Figure 2d–f).

**Figure 8:**
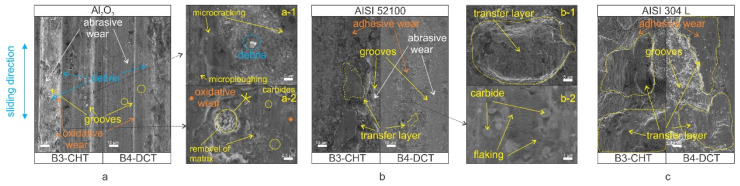
Examples of wear scars for the same wear condition 1 (102 N/15 Hz/0.12 m/s) for (**a**) conventionally heat-treated sample and (**b**) deep cryogenic heat-treated sample. (**a**,**a-1**,**a-2**) SEM micrographs of B3-CHT (**left**) and B4-DCT (**right**) of sample under mentioned conditions with counter-body Al_2_O_3_. (**b**,**b-1**,**b-2**) SEM micrographs of B3-CHT (**left**) and B4-DCT (**right**) of sample under mentioned conditions with counter-body AISI 52100. (**c**) SEM micrographs of B3-CHT (**left**) and B4-DCT (**right**) of sample under mentioned conditions with counter-body AISI 304L.

In wear testing, which favors abrasive wear (Al_2_O_3_ counter-ball), abrasive wear is combined with surface oxidation and oxidative wear (dark grey areas in Figure 8, Supplementary Material Figure S1), mainly observed in the carbide–matrix boundary during high sliding speed conditions. Regarding this aspect, an additional improvement is expected from DCT since the oxide development by the high flash temperatures is reduced, due to a higher amount of carbides and a refined lath structure of the matrix that decreases the amount of absorbed oxygen and build-up of the oxide layer [21]. An important factor also has the mean carbide size as suggested by Vardavoulias 1994 [25]. The carbides, whose mean size is less than or equal to the oxide layer thickness (M_23_C_6_), are incorporated into the densified and hardened surface zone. Overtime, the hardened zone leads to reduced wear of the matrix, being more pronounced for DCT treatment and intensifying the precipitation of very fine M_23_C_6_ carbides.

The testing against an AISI 304L counter ball promoted adhesive wear combined with oxidative wear (Figure 8(b-1,c)). For all steels, heat treatment and contact conditions applied a high degree of AISI 304L counter ball wear, and transfer layers formation took place. Again, for a high hardness case (high austenitizing/low tempering conditions) and matrix properties defining adhesive wear resistance, DCT shows no distinctive effect. However, for a higher toughness case (low austenitizing/high tempering temperature) and the promotion of carbides precipitation with a lower adhesion tendency, DCT has an evidently positive effect, being the most evident for steel C (AISI M35), followed by steel B (AISI M3:2) and A (AISI M2). In the case of steel C, DCT most evidently facilitates the precipitation of finer and more homogeneously distributed carbides, with the matrix material being more segmented by the homogeneously distributed carbides and solid solution hardened with Co, thus reducing adhesion on a macro scale, as also indicated by a decrease in friction. Steel A shows lower volume fraction of carbides while PM steel B already has a very homogeneous carbides distribution, reducing the positive effect of DCT.

In the case of a mixture of abrasive and adhesive wear (AISI 52100 counter-ball), wear grooves are combined with the formation of transfer layers and flaking (Figure 8(b-2)), which additionally intensify the wear of the material. Compared to pure abrasive wear (Al_2_O_3_ counter-ball) DCT generally improves the combined abrasive/adhesive wear resistance of HSS for a high hardness case (high austenitizing/low tempering temperatures). Increased precipitation and the more homogeneous distribution of carbides reduces the adhesive component with a harder matrix being less prone to sticking and tearing. However, the promoted toughness depends on the steel grade, having a similar effect on pure abrasive wear for steel A, a predominantly negative effect for steel B and a predominantly positive effect for steel C. In the case of steel A, displaying the highest hardness and the lowest fraction of carbides, abrasive wear predominates, thus showing the same effect of DCT as for pure abrasive wear conditions. In the case of steel C with the finest carbides and Co, the strengthened matrix-adhesive wear dominates, which is greatly reduced by DCT, while for steel B, a high-volume fraction of carbides and increased toughness increase the likelihood of the material sticking and cold welding to the counter ball, which is then followed by the localized tearing of the surface and intensified wear.

### 4.2. Galling Resistance

**Figure 9:**
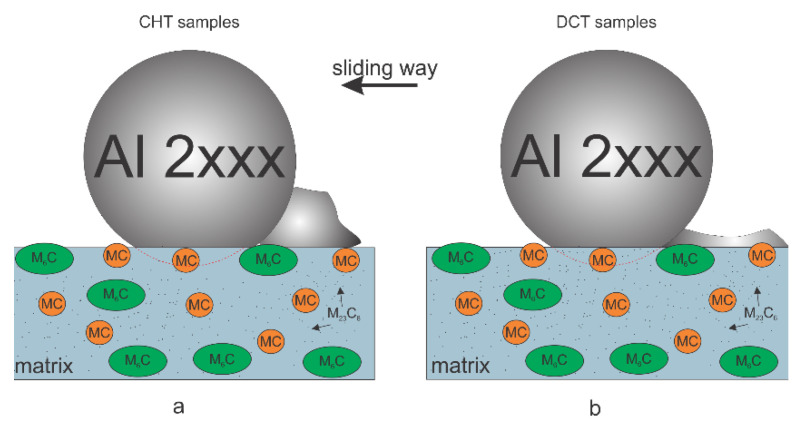
Schematic representation of galling testing of steels corresponding to (**a**) conventional samples and (**b**) deep cryogenic heat-treated samples.

In the case of the galling tests, a generally positive effect of DCT is observed (Supplementary Material Figure S2). The improvement of galling resistance with DCT, similar for adhesive wear resistance, is considered to be a consequence of microstructure and hardness changes. The DCT leads to an increase in M_23_C_6_ carbides precipitation, which allows for the development of a hardened surface that resists localized branching and tearing that would normally result in higher galling. Furthermore, the more even distribution of carbides leads to a more homogeneous and even surface and reduces local surface roughness (confirmed by a previous study [21]). Regarding this aspect, the residual stresses of the surface can be a potentially defining parameter, since the material can produce a higher cracking of the surface with normal loads, when internal tensile stresses are present. Based on previous studies [8,26], it is confirmed that DCT generally reduces the tensile stresses or even changes them into a compressive character. The galling tests shown in relation to the heat treatment also have a generally higher impact on DCT in the case of lower austenitizing and higher tempering temperatures, which also yield a considerably lower friction coefficient and lower adhered volume for all HSS with DCT. This is believed to occur from the much more refined microstructure, due to the smaller prior austenite grains, smaller martensitic lath structure and larger carbide amount compared to the opposite heat treatment [17], which creates a more durable and homogeneous surface that more strongly resists the adhesion and agglomeration of the counter body material (Figure 9a,b). For the treatment with a higher austenitizing and lower tempering temperature, the friction coefficient is generally higher for DCT than for the CHT samples, which are considered to be related to the general higher toughness of the matrix material after DCT. However, as the galling test is in a single stroke configuration and the aluminum material is much softer, this does not directly affect galling intensity. Both DCT and CHT samples from this heat treatment group (groups 1–2) display similar deposited volumes at various, selected, normal load values (Figure 5a–c). Only steel C, with a considerable amount of Co shows a tendency towards a lower volume deposition with DCT. Steel C also sees a more linear increase in COF than steels A and B. This is considered to be related to the solid solution hardening of the matrix with Co that is not depleted from the matrix by carbide precipitation. As a result, such a matrix state, in combination with the increased toughness and higher number of carbides (volumetrically similar), leads to a linear change of COF with an applied load, as well as a linear displacement in friction properties with the application of DCT.

### 4.3. Dynamic Impact Wear

For the impact wear tests, abrasive wear combined with oxidative wear, microcracking and microploughing were dominant for all steels investigated when treated with either CHT or DCT. In all cases, the softer martensitic matrix is worn faster than surrounding carbides, which can be pulled out or microcracked, thus intensifying impact wear. However, a clear correlation with the carbide precipitation can be determined. For all steel grades, a general tendency has a reduced impact wear with the increased volumetric amount and more homogeneous distribution of M_23_C_6_ carbides obtained by DCT. This is concordant with the theory of carbide agglomeration and the subsequent hardening of the matrix of the contact surface, which prevents further wear propagation over time. From this perspective, the increase in impact wear resistance with DCT is also influenced by the increased toughness of the material, since it does not only allow for a deeper movement of carbides into the matrix, but also reduces the probability of the cracking of the matrix with external loading. It should be noted that the increased amount of M_23_C_6_ carbides does not directly relate to an increase in hardness, which results in an inconsistent relationship between hardness and impact wear resistance. However, for steel C, this relation still holds; for this steel the volumetric change in M_23_C_6_ carbides is negligible, which results in a stronger correlation with the hardness/toughness change in the material with different heat treatment procedures that impact wear properties.

## 5. Conclusions

The effect of deep cryogenic treatment (DCT) on sliding wear, impact wear and galling behavior of selected high-speed steels (HSS) (AISI M2, AISI M3:2 and AISI M35) was investigated in correlation with selected austenitization and tempering temperatures and different wear conditions and modes. The DCT effect on sliding wear resistance depended on the HSS steel grade, as well as the contact conditions and wear mode, whereas on dynamic impact wear and galling resistance, DCT had a generally positive effect:

1. The positive effect of DCT on the tribological properties of HSS is attributed to a more homogenous microstructure, an increase in precipitation of finer carbides, the modification of different carbide types, and the generally reduced mean carbide diameter that, consequently, led to better mechanical properties.
2. The eventual negative effect of DCT is related to the combined effect of increased volume fraction of carbides and a tougher matrix, which, under certain contact conditions (combined abrasive/adhesive wear), may lead to the sticking and tearing of the matrix material, and the microcracking and pull-outs of carbides. This exposes carbide–matrix boundaries, which act as the starting locations for oxidative wear, while loose carbides may act as abrasive third-body particles.
3. Through the homogeneous distribution of fine carbides over the contact surface, DCT greatly reduces the adhesion and galling of counter-material, while a tougher matrix and increased volume fraction of carbides provides an improved impact wear resistance of HSS. Under sliding, DCT generally reduces abrasive wear. However, the presence of an adhesive component and carbides pull-out may shift the trend in the opposite direction, especially when combined with milder contact conditions.
4. The selection of the heat treatment regime influences the performance of DCT and, consequently, the changes in the microstructure, mechanical properties and tribological properties of HSS. The most optimal heat treatment regime promoting wear resistance improvement by DCT is hardening from lower austenitizing temperature and high temperature tempering, which promotes carbides precipitation and high toughness over hardness.
5. Alloying elements and steel type influence the type, shape and precipitation of carbides, and thus affect the wear and galling properties. The most positive effect of DCT on wear resistance is obtained for high-alloyed Co-containing steel C (AISI M35), with DCT mainly resulting in the refinement of carbides. At lower alloying element contents (steel A; AISI M2), the effect of DCT diminishes, while, for PM steels with very uniform carbides, the positive effect of DCT becomes a negative effect when abrasive wear is accompanied with adhesion.

## Figures and Tables

**Table 1:** Measured chemical composition of AISI M2, AISI M3:2 and AISI M35 steel (wt. %). Analyzed values measured with ICP-OES Agilent 720.

Steel (AISI)	C	Mn	S	Cr	Mo	W	V	Co	Fe
M2	0.90	0.28	0.002	4.00	4.70	6.00	1.70	-	base
M3:2	1.29	0.31	0.006	3.90	4.80	6.75	3.00	0.69	base
M35	0.90	0.34	0.004	4.10	5.20	6.20	2.01	4.50	base

**Table 2:** Heat treatment of AISI M2, AISI M3:2 and AISI M35 steel samples, where samples 1 and 3, marked as CHT samples, were conventionally heat-treated, and samples 2 and 4, marked as DCT samples, were deep cryogenic heat-treated. In addition to heat treatment parameters, hardness (HRC), fracture toughness (KIc; MPa√m) and impact toughness (KV; J) are provided for each sample subgroup [17].

Steel (AISI)	Subgroup	Austenitizing (°C/min)	DCT (°C/h)	Tempering (°C/h)	HRC	K_Ic_ * (MPa√m)	KV ** (J)
M2 (A)	A1-CHT (A1)	1230/2	-	3x 550/1	66	10	3
	A2-DCT (A2)	1230/2	−196/24	1x 550/1	65	11	3
	A3-CHT (A3)	1180/2	-	3x 620/1	58	12	3
	A4-DCT (A4)	1180/2	−196/24	1x 620/1	60	14	3
M3:2 (B)	B1-CHT (B1)	1180/2	-	3x 540/2	66	10	3
	B2-DCT (B2)	1180/2	−196/24	1x 540/2	65	10	4
	B3-CHT (B3)	1050/2	-	3x 600/2	53	15	4
	B4-DCT (B4)	1050/2	−196/24	1x 600/2	56	12	4
M35 (C)	C1-CHT (C1)	1230/2	-	3x 550/2	66	13	3
	C2-DCT (C2)	1230/2	−196/24	1x 550/2	65	13	4
	C3-CHT (C3)	1160/2	-	3x 620/2	53	13	3
	C4-DCT (C4)	1160/2	−196/24	1x 620/2	57	13	3

* measured by circumferentially notched and fatigue pre-cracked tensile bar specimen [18], ** measured by Charpy V-notch impact test.

**Table 3:** Sliding conditions.

Sliding Condition	Speed (m/s)	Contact Pressure (GPa)
Condition 1 (Al_2_O_3_/AISI 52100/AISI 304L)	0.12	102/40/40
Condition 2 (Al_2_O_3_/AISI 52100/AISI 304L)	0.01	102/40/40
Condition 3 (Al_2_O_3_/AISI 52100/AISI 304L)	0.12	30/20/20
Condition 4 (Al_2_O_3_/AISI 52100/AISI 304L)	0.01	30/20/20

## Data Availability

The raw/processed data required to reproduce these findings cannot be shared at this time as the data also forms part of an ongoing study.

## Supplementary Materials

The following are available online at https://www.mdpi.com/1996-1944/14/24/7561/s1, Supplementary Material Table S1, The phase results for all three high-speed steels; Supplementary Material Figure S1, Oxidation; Supplementary Material Figure S2, Galling testing.
