# Correction: Jovičević-Klug et al. Effect of Deep Cryogenic Treatment on Wear and Galling Properties of High-Speed Steels. *Materials* 2021, *14*, 7561

**DOI:** 10.3390/ma15207218

**Published:** 2022-10-17

**Authors:** Patricia Jovičević-Klug, Marko Sedlaček, Matic Jovičević-Klug, Bojan Podgornik

**Affiliations:** 1 Institute of Metals and Technology, Lepi Pot 11, 1000 Ljubljana, Slovenia; 2 Jožef Stefan International Postgraduate School, Jamova Cesta 39, 1000 Ljubljana, Slovenia; 3 Max-Planck-Institute für Eisenforschung, Max-Planck-Straße 1, 40237 Dusseldorf, Germany

In Sections 3.1.1 and 4.1, we present the results as follows [1]:

“On the other hand, when hardened from a low austenitization temperature (promoting toughness; A3, A4), DCT greatly improves the abrasive wear resistance of steel A under high sliding speed conditions (~75%) but deteriorates it with low sliding speeds (2–3 times). In the case of steel B (k = 0.1 × 10^−6^−5.0 × 10^−6^ mm^3^/Nm), DCT improves its abrasive wear resistance under high sliding speed conditions regardless of the austenitization/tempering temperatures, with the improvement being in the range of 30–60%. However, under low sliding speed conditions in combination with a high austenitization temperature and low tempering temperature, DCT results in a reduced abrasive wear resistance (up to 4 times), while with low austenitization temperature and high tempering temperature followed by DCT causes an improvement of up to 40%, especially for low loading conditions (Figure 2e). Finally, for steel C (k = 1.0 × 10^−6^−35.0 × 10^−6^ mm^3^/Nm) there is a general trend of negligible to only minor negative effectd on abrasive wear resistance, when applying DCT treatment (Figure 2f).”

**Figure 2:**
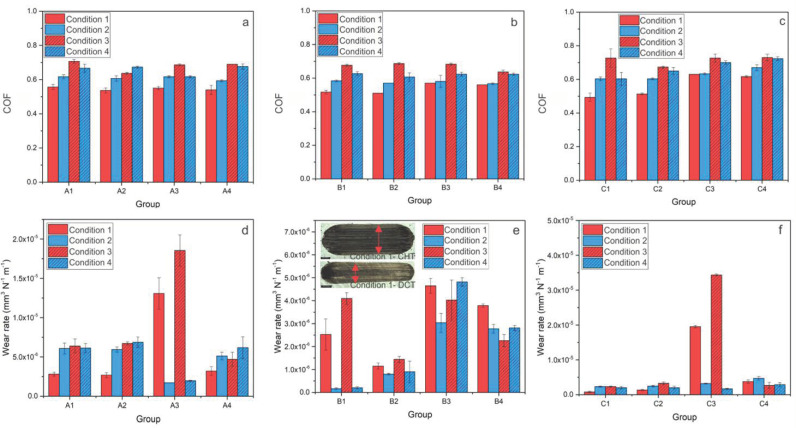
Results of abrasive wear conditions (Al_2_O_3_ counter-body) for all three steels; A (AISI M2), B (AISI M3:2) and C (AISI M35), where (**a**–**c**) is steady-state coefficient of friction for each condition and (**d**–**f**) is wear rate for each steel at certain condition. (**e**) the insert shows the average representation of wear scar for all three steels of conventionally (CHT) and deep cryogenically heat-treated samples, the black scale represents 500 µm.

Section 4.1:

“In terms of the abrasive wear resistance (Figure 7a,b), DCT generally has no dominant effect on heat treatment conditions that favor high hardness (high austenitizing/low tempering temperature), with the matrix hardness being the dominant factor. One exception is PM steel B, which has a higher volume fraction and more uniform carbides. However, under conditions favoring a higher toughness (low austenitizing/high tempering temperature) DCT improves the abrasive wear resistance of HSS steels for high sliding speed conditions, while it decreases it under low-speed conditions. The general tendency of a more pronounced effect of DCT on abrasive wear resistance at lower austenitizing and higher tempering conditions is associated with the softer matrix, higher precipitation of carbides and a higher toughness, also typical for PM steels. In this case, DCT promotes the increased precipitation of finer carbides and their more homogeneous distribution, facilitating improvement in toughness as well as hardness (Table 2). It is proposed that, at high sliding speeds, the increased toughness allows for the localized deformation of the softened matrix material due to high contact temperatures, which, in turn, allow the displacement of the carbides on the contact surface and deeper into the matrix (indicated by vertical red arrows in Figure 7b), protecting the matrix from nominal friction forces and wear. In turn, the initial removal of material mostly results from the accommodation of this effect, which is then considerably reduced with the intensified agglomeration of carbides over time. However, at low sliding speeds, the absence of the matrix softening promotes carbides pull-out (mostly MC carbides, as also observed by Pellizzari et al., 2012 [9]) and microcracking (M_23_C_6_ and M_6_C), which may act as third-body particles and cause microploughing, thus resulting in an increased wear rate at an increased level of carbides precipitation via DCT. This effect strongly depends on the size and shape of carbides. These carbides are smaller and more uniform, with a smaller size having a negative effect, thus switching favor toward DCT, as observed for PM steel B.”

After publication, we noticed an error in the data visualization in Figure 2. Therefore, we have made the following correction to Figure 2:

**Figure 2:**
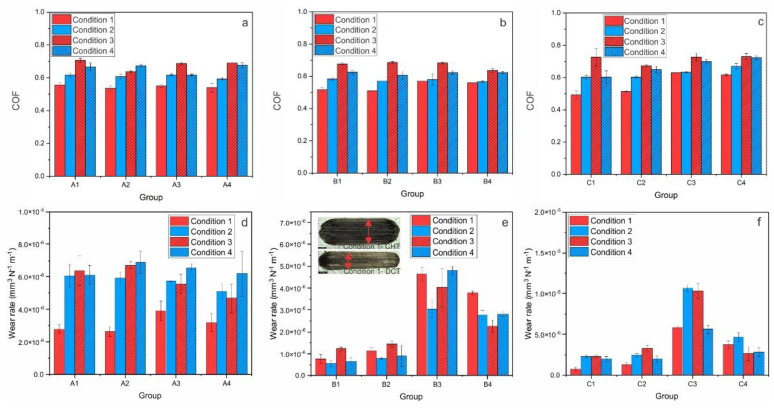
Results of abrasive wear conditions (Al_2_O_3_ counter-body) for all three steels; A (AISI M2), B (AISI M3:2) and C (AISI M35), where (**a**–**c**) is steady-state coefficient of friction for each condition and (**d**–**f**) is wear rate for each steel at certain condition. (**e**) The insert shows the average representation of wear scar for all three steels of conventionally (CHT) and deep cryogenically heat-treated samples, the black scale represents 500 µm.

Consequently, the second half of the paragraph text in Sections 3.1.1 and 4.1. second paragraph, which are associated with Figure 2, were also updated accordingly:

“On the other hand, when hardened from low austenitization temperature (promoting toughness; A3, A4), generally DCT improves abrasive wear resistance of steel A regardless of the sliding condition (up to 25%). In the case of steel B (k = 0.5 × 10^−6^−5.0 × 10^−6^ mm^3^/Nm), DCT generally improves its abrasive wear resistance under low austenitization temperature and high tempering temperature regardless of the sliding conditions, with the improvement being in the range of 30–60%, especially prominent for low loading conditions (Figure 2e). However, under all sliding conditions in combination with high austenitization temperature and low tempering temperature, DCT results in reduced abrasive wear resistance (up to 20%). Finally, for steel C (k = 1.0 × 10^−6^−11.0 × 10^−6^ mm^3^/Nm) there is a general trend of negligible to only minor negative effect on abrasive wear resistance, when applying DCT treatment in combination with high austenitization temperature and low tempering temperature (Figure 2f). Whereas with low austenitization temperature and high tempering temperature, DCT improves the wear resistance regardless of sliding condition, up to 50%.”

Section 4.1:

“In terms of the abrasive wear resistance (Figure 7a,b), in general DCT has no dominant effect for heat treatment conditions favoring high hardness (high austenitizing/low tempering temperature), with the matrix hardness being the dominant factor. However, more homogeneous and finer carbides distribution with reduced agglomeration may even result in minor reduction of abrasive wear resistance after DCT. However, under conditions favoring higher toughness (low austenitizing/high tempering temperature) DCT improves abrasive wear resistance of HSS steels for all contact conditions, especially for steel B and C, also displaying considerable increase in hardness. The general tendency of more pronounced effect of DCT on abrasive wear resistance at lower austenitizing and higher tempering conditions is associated with the softer matrix, higher precipitation of carbides and higher toughness, also typical for PM steels. In this case, DCT promotes increased precipitation of finer carbides and their more homogeneous distribution, facilitating improvement in toughness as well as hardness (Table 2). It is proposed that the increased toughness allows the localized deformation of the softened matrix material due to high contact temperatures, which in turn allows displacement of the carbides on the contact surface and deeper into the matrix (indicated by vertical red arrows in Figure 7b), protecting the matrix from the nominal friction forces and wear. In turn, the initial removal of material mostly results from the accommodation of this effect, which is then considerably reduced with intensified agglomeration of carbides over time. At low sliding speeds absence of matrix softening could promote carbides pull-out (mostly MC carbides, as also observed by Pellizzari et al., 2012 [9]) and microcracking (M_23_C_6_ and M_6_C) which may act as third body particles and causing microploughing, thus resulting in increased wear rate at increased carbides precipitation by DCT. This effect strongly depends on contact conditions and the size and shape of carbides. Smaller and more uniform, smaller is the negative effect, thus switching favor toward DCT, as observed in our case (see Figure 2d–f).”
