# Supplementary material for: Effect of Deep Cryogenic Treatment on Wear and Galling Properties of High-Speed Steels
Source: Materials (Basel). 2021 Dec 9;14(24):7561. doi: 10.3390/ma14247561 (PMC8705678; doi:10.3390/ma14247561)

materials

MDPI

*Article*

# Effect of Deep Cryogenic Treatment on Wear and Galling Properties of High-Speed Steels

**Patricia Jovičević-Klug [1,2,*], Marko Sedlaček [2], Matic Jovičević-Klug [3] and Bojan Podgornik [1,2]**

[1] Institute of Metals and Technology, Lepi pot 11, 1000 Ljubljana, Slovenia; bojan.podgornik@imt.si
[2] Jožef Stefan International Postgraduate School, Jamova cesta 39, 1000 Ljubljana, Slovenia; marko.sedlacek@imt.si
[3] Max-Planck-Institute für Eisenforschung, Max-Planck-Straße 1, 40237 Düsseldorf, Germany; m.jovicevic-klug@mpie.de
* Correspondence: patricia.jovicevicklug@imt.si; Tel.: +386-1-4701-990

**Abstract:** New approaches in improving wear resistance are getting attention, with an affordable and noncomplex technology, such as deep cryogenic treatment (DCT). The aim of this study is to investigate the effect of DCT on friction and wear performance of high-speed steels. AISI M2, AISI M3:2 and AISI M35 were heat-treated under different conditions and then investigated under dry sliding conditions. Tribological testing involved different contact conditions, prevailing wear mechanisms and loading conditions. DCT effect on sliding wear resistance depends on HSS steel grade as well as contact conditions and wear mode, whereas it improves dynamic impact wear and galling resistance.

**Keywords:** deep cryogenic treatment; high speed steel; wear; galling; impact loading

**Supplementary Material Table S1**. The phase results for all three high-speed steels.

| Steel subgroup | Carbides | | | Matrix |
|---|---|---|---|---|
| | MC (V) | $M_6C$ (W, Mo, Fe) | $M_{23}C_6$ (Fe, Cr) | |
| **A1-CHT** | 2.1 | 6.9 | 2.2 | 88.8 |
| A2-DCT | 1.5 | 6.7 | 5.3 | 86.3 |
| A3-CHT | 1.8 | 6.7 | 2.5 | 89.1 |
| A4-DCT | 1.6 | 6.8 | 6.8 | 84.8 |
| B1-CHT | 6.6 | 7.6 | 4.3 | 81.5 |
| B2-DCT | 5.9 | 7.2 | 7.7 | 79.2 |
| B3-CHT | 7.5 | 8.4 | 3.1 | 81.1 |
| B4-DCT | 7.5 | 9.3 | 8.2 | 75.1 |
| C1-CHT | 3.0 | 7.3 | 5.1 | 84.6 |
| C2-DCT | 3.1 | 7.6 | 4.9 | 84.4 |
| C3-CHT | 2.8 | 7.1 | 3.4 | 86.7 |
| C4-DCT | 3.2 | 8.4 | 3.6 | 84.8 |

**Supplementary Material Figure S1.** Oxidation.

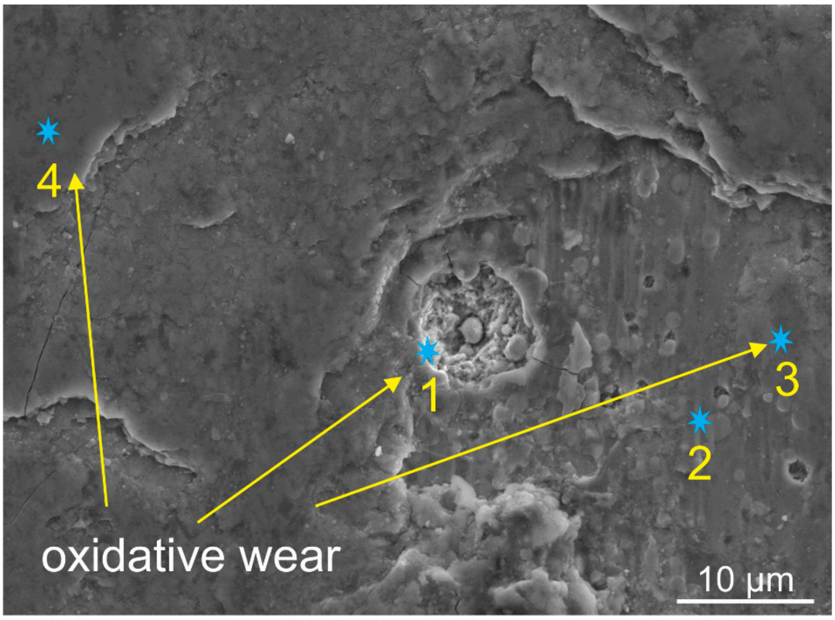


| Spectrum | C | O | Al | Si | V | Cr | Mo | W | Fe |
|---|---|---|---|---|---|---|---|---|---|
| Spectrum 1 | 35.84 | 19.47 | 0.13 | 0.62 | 2.08 | 3.53 | 9.27 | 6.56 | base |
| Spectrum 2 | 21.08 | 9.26 | 0.35 | 1.47 | 3.38 | 3.92 | 15.51 | 12.07 | base |
| Spectrum 3 | 12.69 | 55.77 | 0.25 | 0.38 | 1.18 | 1.59 | 1.13 | 0.75 | base |
| Spectrum 4 | 11.10 | 33.37 | 0.24 | 0.84 | 0.78 | 2.70 | 0.99 | 0.63 | base |

**Supplementary Material Figure S2.** Galling testing.

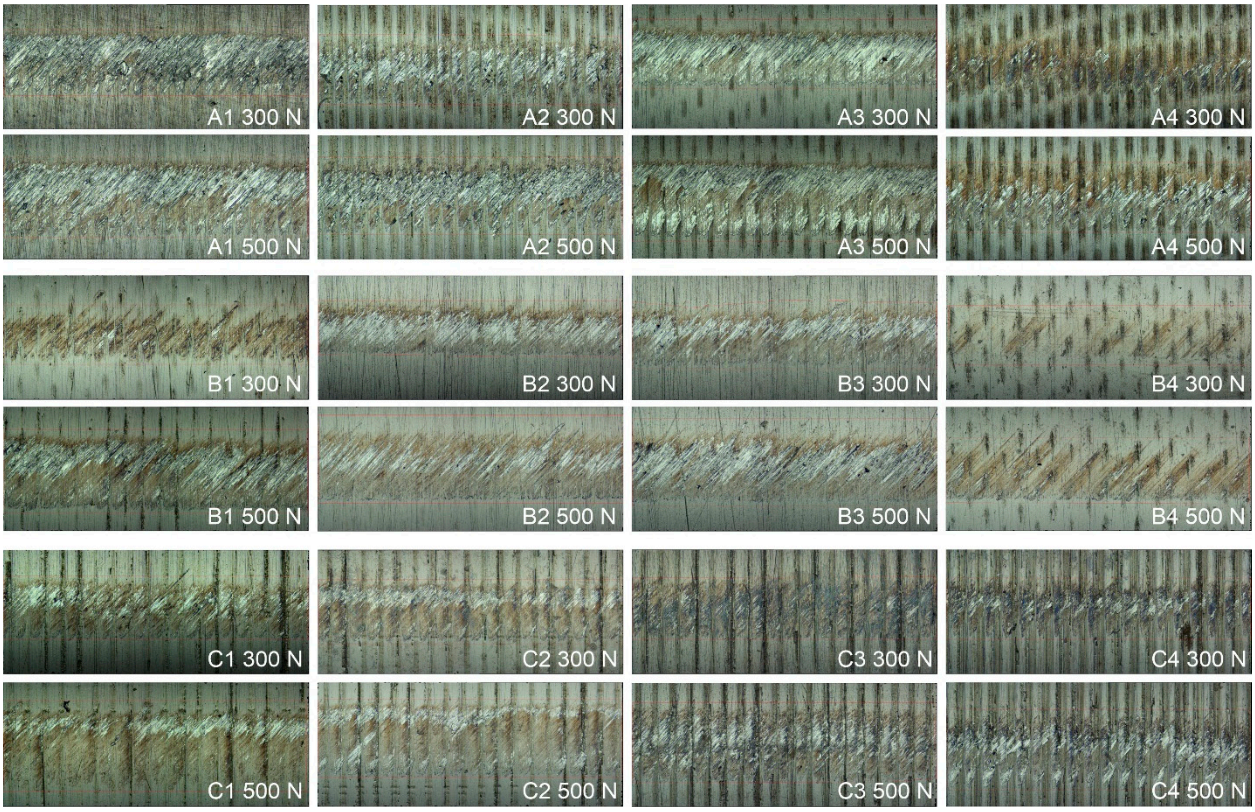

Supplement: Supplementary file 1 [file materials-14-07561-s001.zip › materials-1491507-supplementary.pdf]
